# The fully synthetic MAG-Tn3 therapeutic vaccine containing the tetanus toxoid-derived TT830-844 universal epitope provides anti-tumor immunity

**DOI:** 10.1007/s00262-016-1802-0

**Published:** 2016-02-04

**Authors:** Daphné Laubreton, Sylvie Bay, Christine Sedlik, Cécile Artaud, Christelle Ganneau, Edith Dériaud, Sophie Viel, Anne-Laure Puaux, Sebastian Amigorena, Catherine Gérard, Richard Lo-Man, Claude Leclerc

**Affiliations:** 1 Unité de Régulation Immunitaire et Vaccinologie, Equipe Labellisée Ligue Contre le Cancer, Institut Pasteur, 25 rue du Docteur Roux, 75015 Paris, France; 2 Institut National de la Santé et de la Recherche Médicale U1041, Paris, France; 3 Unité de Chimie des Biomolécules, Institut Pasteur, Paris, France; 4 Centre National de la Recherche Scientifique UMR3523, Paris, France; 5 Institut Curie, Paris Sciences et Lettres Research University, Paris, France; 6 Institut National de la Santé et de la Recherche Médicale U932, Paris, France; 7 Pôle Intégré de Recherche Clinique, Institut Pasteur, Paris, France; 8 GSK Vaccines, Rixensart, Belgium

**Keywords:** Universal epitope, TT830-844, MAG-Tn3, Antibody response, Anticancer vaccine

## Abstract

Malignant transformations are often associated with aberrant glycosylation processes that lead to the expression of new carbohydrate antigens at the surface of tumor cells. Of these carbohydrate antigens, the Tn antigen is particularly highly expressed in many carcinomas, especially in breast carcinoma. We designed MAG-Tn3, a fully synthetic vaccine based on three consecutive Tn moieties that are *O*-linked to a CD4^+^ T cell epitope, to induce anti-Tn antibody responses that could be helpful for therapeutic vaccination against cancer. To ensure broad coverage within the human population, the tetanus toxoid-derived peptide TT830-844 was selected as a T-helper epitope because it can bind to various HLA-DRB molecules. We showed that the MAG-Tn3 vaccine, which was formulated with the GSK proprietary immunostimulant AS15 and designed for human cancer therapy, is able to induce an anti-Tn antibody response in mice of various H-2 haplotypes, and this response correlates with the ability to induce a specific T cell response against the TT830-844 peptide. The universality of the TT830-844 peptide was extended to new H-2 and HLA-DRB molecules that were capable of binding this T cell epitope. Finally, the MAG-Tn3 vaccine was able to induce anti-Tn antibody responses in cynomolgus monkeys, which targeted Tn-expressing tumor cells and mediated tumor cell death both in vitro and in vivo. Thus, MAG-Tn3 is a highly promising anticancer vaccine that is currently under evaluation in a phase I clinical trial.

**Electronic supplementary material:**

The online version of this article (doi:10.1007/s00262-016-1802-0) contains supplementary material, which is available to authorized users.

## Introduction

Malignant transformations are often associated with a deregulation of glycosylation processes, which leads to the expression of tumor-associated carbohydrate antigens at the surface of tumor cells [1]. The Tn antigen, defined as an α-D-N-acetylgalactosamine (GalNAc) linked to a serine or threonine residue, is one of the most tumor-specific carbohydrate antigens; it is expressed in approximately 90 % of carcinomas [2], including 80 % of breast cancer tissues [3]. These attributes make Tn an interesting target for the development of anticancer vaccines.

Carbohydrate antigens are T-cell-independent antigens [4]. Because they are not able to induce an efficient antibody response by themselves, they need to be linked to appropriate carrier molecules. Therefore, we developed a dendrimeric multiple antigenic glycopeptide (MAG), based on a multivalent display of a Tn antigen trimer associated with a T-helper epitope, to induce a specific anti-Tn antibody response. We previously demonstrated the efficacy of the MAG strategy in mice using the poliovirus (PV) peptide as a T-helper epitope. Indeed, the MAG-Tn3-PV construct was able to induce high levels of anti-Tn antibodies in BALB/c mice and, more importantly, led to the survival of 80 % of mammary tumor-bearing mice (TA3Ha) after either prophylactic or therapeutic treatment [5, 6].

One major obstacle to vaccination in humans is the high degree of MHC polymorphism in the human population; more than 12,000 class I and class II MHC alleles have been reported so far [7]. However, broad coverage within the human population can potentially be achieved by using a promiscuous T-helper epitope, such as the tetanus toxoid-derived P2 peptide TT830-844 (TT). TT has been described to be universally immunogenic in various species, including mice [8–10] and humans [8, 11–15].

Thus, we designed a MAG-Tn3 vaccine for active immunotherapy in humans, using TT as a T-helper epitope (Sup. Fig. 1), and analyzed in the present study its capacity to induce anti-tumor responses in preclinical models. We demonstrated its ability to induce high anti-Tn antibody levels in both mice and non-human primates. Furthermore, we confirmed and extended our knowledge about murine and human MHC II molecules that are able to bind TT. Finally, we demonstrated the anti-tumor potential of the anti-Tn antibodies induced in response to MAG-Tn3 vaccination and showed their capacity to recognize and mediate killing of tumor cells in vitro and in vivo.

The present study thus established that the MAG-Tn3 containing the TT epitope, produced under good manufacturing practices (GMP) condition and formulated in AS15, is able to induce anti-Tn antibodies with a high therapeutic potential in various preclinical models. Based on these results, the MAG-Tn3/AS15 is currently under evaluation as a therapeutic vaccine for breast cancer patients, in a phase I clinical trial.

## Materials and methods

### Mice

HLA-DR1*A2 transgenic mice expressing human HLA-DRB1*01:01, HLA-DRA*01:01 and HLA-A*02:01 [16] were from the Pasteur Institute animal colony. Six-week-old female C57BL/6J, BALB/c, C3H/HeN, NMRI and CD-1 mice were purchased from Charles River Laboratories. Mice were kept in the Pasteur Institute animal house and supplied with water and food ad libitum. All procedures involving mice were in accordance with the French Ethical Committee CETEA (comité d’éthique en expérimentation animale) (Project No. 2013-126).

### Non-human primates

Cynomolgus monkeys (20 per gender) were bred and quarantined at Le Tamarinier (Tamarin, Mauritius) before being shipped to CiToxLAB France at 27–32 months old. Primates were acclimated to the study conditions for a period of 15 days before beginning treatment. All animals had free access to tap water, and food was distributed daily. The study was performed in compliance with CiToxLAB France’s standard operating procedures and the principles of Good Laboratory Practice, and the study was in compliance with Animal Health regulations.

### Cells and reagents

The Tn-expressing Jurkat cell line transfected with GFP (Jurkat-GFP) [17], mammary carcinoma cell line MCF-7 [18], ovarian carcinoma cell lines OVCAR [19] and SHIN3 [20], and the murine mammary carcinoma TA3Ha cell line [21] were cultured in complete RPMI 1640 medium (Invitrogen) supplemented with 10 % FCS (Hyclone) and antibiotics (100 U/mL penicillin and 100 μg/mL streptomycin, Invitrogen).

The MAG-Tn3 vaccine was produced by Lonza as previously described [22]. AS15 is a combination of AS01B and CpG 7909 synthetic oligodeoxynucleotides (ODNs) containing unmethylated CpG motifs in a liposomal formulation [23]. The MAG-Tn3/AS15 formulation consisted of lyophilized MAG-Tn3 (300 μg) combined with CpG (420 µg) and reconstituted with the AS01B adjuvant (500 μL) at the time of administration.

Complete and incomplete Freund’s adjuvants (respectively, CFA and IFA) were purchased from Sigma-Aldrich. The tetanus toxoid TT830-844 peptide (QYIKANSKFIGITEL) and the H-2^k^-restricted HEL45-61 peptide derived from hen egg lysozyme (RNTDGSTDYGILQINSR) [24] were obtained from Polypeptide.

The anti-CD4 (GK1.5) monoclonal antibody (mAb) was prepared from ascitic fluid [25]. The anti-rat IgG2b isotype control (LTF2) was purchased from BioXCell. The IgG2b murine anti-Tn mAb 8D4 was obtained from BALB/c mice immunized with MAG-Tn3-PV, as previously described [3]. The Herceptin mAb (trastuzumab, anti-Her2) was purchased from Hoffmann-La Roche. The chimeric Chi-Tn mAb was produced as previously described [26].

### Immunization

For the vaccination experiment, mice were intramuscularly (im) immunized with either 1/20, 1/7 or 1/5 of MAG-Tn3 formulated with AS15, which corresponds to 15, 45 or 60 μg of MAG-Tn3. Immunizations were performed 3–5 times at 3-week intervals.

For the T cell depletion experiment, mice vaccinated on days 0 and 21 were intraperitoneally (ip) treated with anti-CD4 or isotype control antibody (300 μg) on days −1, 0, 1, 20, 21 and 22.

For the peptide presentation experiment, mice were subcutaneously (sc) immunized with 50 μg of either TT or HEL formulated in CFA. In some experiments, a second injection was performed at day 11 with peptide formulated in IFA.

Cynomolgus monkeys were immunized with NaCl or AS15 alone (sc) or 300 μg of MAG-Tn3 formulated with the AS15 immunostimulant (sc or im). Immunizations were performed 7 times at 3-week intervals.

### Measurement of Tn-specific antibody response by ELISA and flow cytometry

Sera from immunized mice and monkeys were tested for the presence of anti-Tn antibodies by ELISA, using neutravidin pre-coated plates (Thermo Scientific) coated with the synthetic biotinylated Tn3-G6K(Biot)G glycopeptide [6]. The detection antibodies anti-mouse-IgM-HRP and anti-mouse-IgG-HRP or anti-human-IgM-HRP and anti-human-IgG-HRP (Sigma-Aldrich) were used. The antibody titers were calculated as previously described [5].

Cynomolgus sera were also tested for their ability to bind to the Tn-expressing human cell lines using goat antihuman-IgM-FITC and IgG-PE (Southern Biotech). Antibody titers were calculated as previously described [6].

### Measurement of T cell response by intracellular staining

Mouse cell suspensions were stimulated with 50 μg/mL of TT or HEL peptide in the presence of BD GolgiPlug™ (BD Biosciences) for 2 h (37 °C). Cells were fixed and stained using the BD Cytofix/Cytoperm™ Kit (BD Biosciences). Surface and intracellular staining (ICS) were performed according to the manufacturer’s instructions, with anti-CD4-FITC, anti-CD3-APC-eFluor^®^780, anti-CD19-PECy7, anti-TNF-α-PerCP-e710, anti-IL-2-APC and anti-IFNγ-EF450 from eBioscience and anti-CD8a-PE from BD Biosciences.

### Measurement of T cell response by ELISA and ELISPOT

The murine T cell response was analyzed by ELISA, using antibodies against IFN-γ and IL-5 (BD Biosciences), IL-13 and IL-17 (R&D System). Measurements were performed on culture supernatants of mouse cell suspensions stimulated with or without 50 μg/mL of TT for 48–72 h. The cynomolgus IFN-γ response was analyzed on PBMC stimulated for 48 h with 50 μg/mL of TT using the monkey IFN-γ ELISpot^BASIC^ (ALP) kit from Mabtech, according to the manufacturer’s instructions. Spot-forming cells (SFC) were counted using the ELISPOT reader Bioreader^®^ 5000 E-β (Biosys). All measurements were performed in triplicate.

### Complement-dependent cytotoxicity assay

The complement-dependent cytotoxicity assay (CDC) was performed with IgG purified from pre-immunized (day 1) and post-immunized (day 126) sera of cynomolgus monkeys using Zeba™ Spin Desalting Columns (Thermo Scientific) and the Melon Gel IgG Purification Kit (Thermo Scientific). CDC activity was evaluated in Jurkat-GFP cells and MCF-7, SHIN3 and OVCAR cells labeled with 5 μM of CFSE (carboxyfluorescein succinimidyl ester, Life Technologies). Cells (6000/well) were incubated with cynomolgus antibodies at various concentrations and DRAQ7 viability dye (Abcam) at 2 μM, in the presence or absence of 1 % of rabbit complement-MA (Cedarlane) for 2 h in 384-well plates (37 °C). Green fluorescence (live cells) and red (dead cells) fluorescence were measured on an Incucyte™ ZOOM instrument (Essen Bioscience), and the number of cells was calculated using ZOOM software (Essen Bioscience). All measurements were performed in duplicate. The percentage of CDC was determined as follows: % of CDC = ((% of dead cells)^complement^ – (% of dead cells)^medium^) normalized against the control without antibody.

### In vivo tumor immunotherapy

Mice were injected ip on day 0 with 10^3^ TA3Ha tumor cells. The day after the graft (day 1), mice were injected ip with cyclophosphamide (CTX) at 50 mg/kg. Starting from day 2 after the graft, mice were treated twice a week with 8D4, the Chi-Tn anti-Tn mAb [26] or Herceptin (trastuzumab) at 20 mg/kg ip (six total injections). Mouse survival was monitored over a 1.5-month period.

### HLA binding assay

The binding kinetics of TT for 24 human HLA-DRB molecules were measured using the REVEAL^®^MHC binding assay (ProImmune; www.proimmune.com). Detection of bound peptide was based on the presence or absence of the native conformation of the HLA–peptide complex, which is detected by a specific monoclonal antibody. The score of the test peptide is reported quantitatively as a percentage of the signal generated by the positive control peptide. Scores >14 were considered positive for binding.

### Statistical analysis

Statistical analyses were performed using parametric Student’s *t* tests (GraphPad Software). *P* values <0.05 were considered statistically significant. Kaplan–Meier survival curves were created using the log-rank test on GraphPad Software.

## Results

### Immunogenicity of the MAG-Tn3 vaccine in mice with different H-2 haplotypes

To ensure broad coverage of the MAG-Tn3 vaccine within the human population, we selected the universal TT830-844 peptide (TT) as a T-helper epitope. The AS15 immunostimulant, already accepted for human use [27], was selected for clinical evaluation based on its ability to induce high anti-Tn antibody titers in HLA-DR1*A2 mice expressing the human HLA-DRB1*01:01 allele, known to bind TT [8, 11, 13, 28, 29] (Sup. Fig. 2). The clinical formulation of MAG-Tn3 with the AS15 immunostimulant, produced under GMP conditions, was evaluated in mice.

**Fig. 1:**
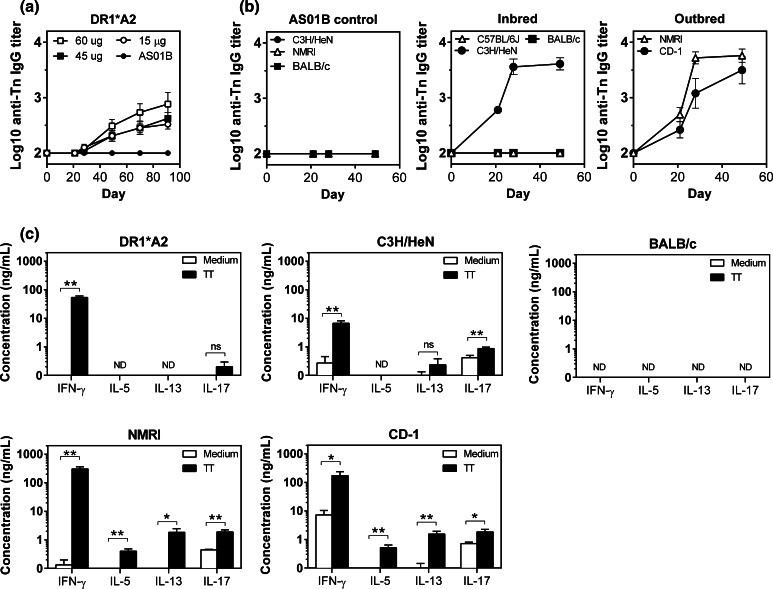
Immunizing mice of different H-2 haplotypes with MAG-Tn3 induce a specific anti-Tn IgG response associated with a TT-specific T cell response. **a** HLA-DR1*A2 transgenic mice were im immunized on days 0, 21, 42, 63 and 84 with 15 (*n* = 12), 45 (*n* = 12) or 60 μg (*n* = 10) of MAG-Tn3 with 1/20, 1/7 and 1/5 of the AS15 immunostimulant, respectively, or with the AS01B immunostimulant alone. **b** C57BL/6J, C3H/HeN, BALB/c mice (*n* = 6) and NMRI and CD-1 mice (*n* = 8) were im immunized on days 0, 21 and 42 with 15 μg of MAG-Tn3 with 1/20 of the AS15 immunostimulant (*middle* and *right panels*), or with the AS01B immunostimulant alone (*left panel*). Sera were collected on days 21, 28 and 49 and tested for Tn-specific IgG by ELISA using Tn3-G6K (Biot) G. Antibody titers are expressed as the mean of Log10 individual antibody titers ± SEM. **c**. IFN-γ, IL-5, IL-13 and IL-17 production was analyzed by ELISA on supernatants of splenocytes stimulated with (*black bars*) or without (*white bars*) 50 μg/mL of TT for 72 h. The results are expressed as the means of individual mice ± SEM. The statistical significance of differences was determined by the Student *t* test (*P < 0.05, **P < 0.01). *ND* not detectable

The immunogenicity of MAG-Tn3 formulated with AS15 was first tested on HLA-DR1*A2 mice, and similar anti-Tn IgG titers were observed at the three MAG-Tn3 doses tested (Fig. 1a). The lowest dose (15 μg) was selected for further experimentation.

The immunogenicity of the MAG-Tn3/AS15 formulation was tested in several inbred mouse strains, C3H/HeN (H-2^k^), C57BL/6 J (H-2^b^) and BALB/c (H-2^d^) and outbred NMRI and CD-1 mice. Following three immunizations, no anti-Tn IgG production was detected in C57BL/6J and BALB/c sera, while C3H/HeN and both outbred mice NMRI and CD-1 produced high amounts of anti-Tn IgG (Fig. 1b), demonstrating the ability of the TT peptide to help the production of anti-Tn antibodies in mice expressing various H-2 haplotypes.

We then evaluated the T cell response to TT after MAG-Tn3 vaccination by measuring the production of T_H1_, T_H2_ and T_H17_ cytokines in response to in vitro stimulation with TT (Fig. 1c). Because the binding of TT to the H-2^d^ molecule has been previously described [9, 10], we also evaluated the T cell response induced in BALB/c mice. No cytokine production was detected for BALB/c mice, whereas IFN-γ was found to be the main cytokine produced in response to TT in all other strains of mice. An IL-17 response was also observed in C3H/HeN, and IL-17, IL-5 and IL-13 were detected in NMRI and CD-1. The anti-Tn antibody production induced in response to MAG-Tn3 vaccination is therefore associated with a T cell response to TT, mainly oriented toward a T_H1_ profile.

**Fig. 2:**
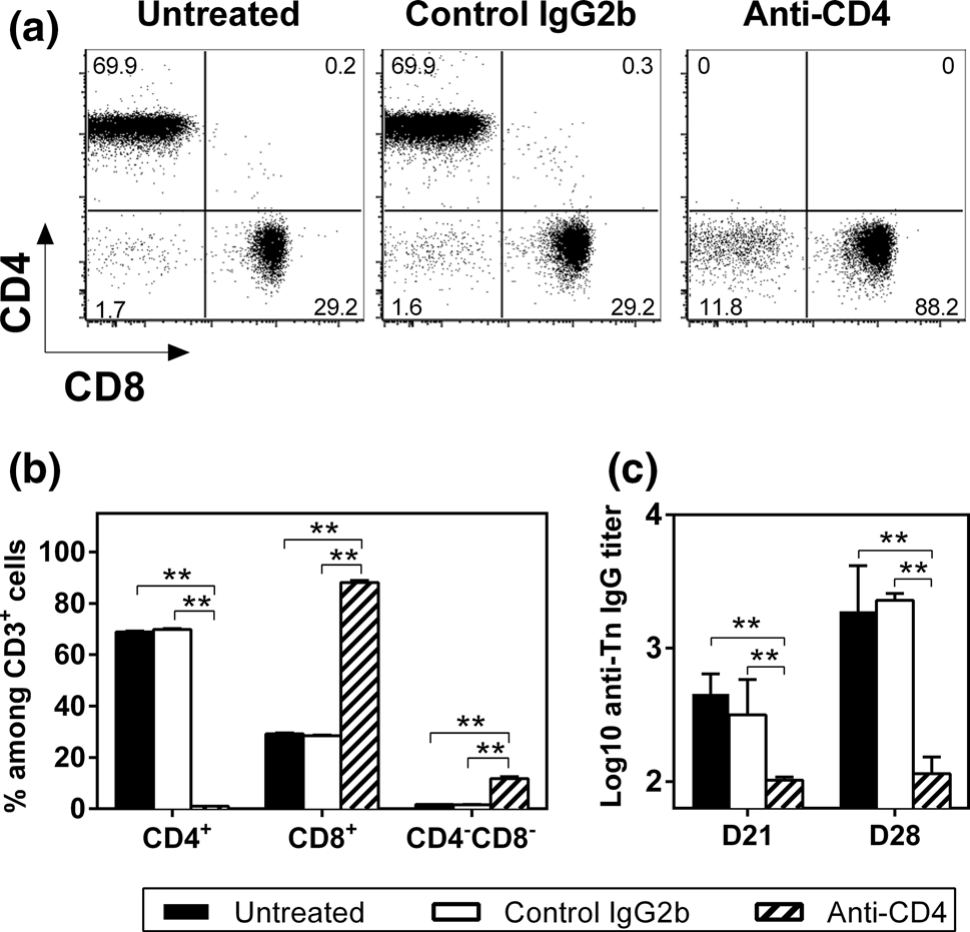
The anti-Tn IgG response induced by the MAG-Tn3 vaccine is CD4^+^ T cell dependent. **a**, **b** C3H/HeN mice (*n* = 6/group) that were left untreated or treated with anti-CD4 or isotype control antibodies (300 μg ip) on days −1, 0, 1, 20, 21 and 22 were im immunized on days 0 and 21 with 15 μg of MAG-Tn3 with 1/20 of the AS15 immunostimulant. CD4^+^ T cell depletion was confirmed by FACS analysis of the percentages of CD4^+^ and CD8^+^ cells among CD3^+^ cells on blood samples collected on day 21, prior to vaccination. *Dot plots* of one representative mouse per group are shown (**a**). The results are expressed as the mean of individual mice ± SEM (**b**). **c** Sera were collected on days 21 and 28 and tested for Tn-specific IgG by ELISA, using Tn3-G6K (Biot) G. Antibody titers are expressed as the mean of Log10 individual antibody titers ± SEM. The statistical significance of differences was determined by the Student *t* test (**P < 0.01)

We previously demonstrated that in the absence of a T-helper epitope, MAG-Tn3 is not able to induce anti-Tn antibody production in mice [30]. To confirm the CD4^+^ T cell dependency of the anti-Tn antibody response, we measured the immunogenicity of the MAG-Tn3 vaccine with AS15 in C3H/HeN mice depleted of CD4^+^ T cells. As shown in Fig. 2a, b, no CD4^+^ T cells were detectable in the blood of anti-CD4-treated mice harvested at day 21, confirming the complete depletion of CD4^+^ T cells at the time of vaccination. As expected, no anti-Tn IgG production was induced in these mice, whereas untreated mice or mice treated with the control isotype produced high amounts of anti-Tn IgG (Fig. 2c), confirming the CD4^+^ T cell requirement for anti-Tn antibody production by B cells.

### Immunogenicity of TT in mice of different H-2 haplotypes

We demonstrated the ability of TT to induce a T cell response to the MAG-Tn3 vaccine in mice expressing various H-2 haplotypes. To confirm the promiscuity of TT in mice, both inbred (C3H/HeN, C57BL/6J and BALB/c) and outbred (NMRI and CD-1) mice were directly immunized with TT in CFA, and cytokine production was measured by ICS and ELISA. HLA-DR1*A2 mice were used as a positive control.

**Fig. 3:**
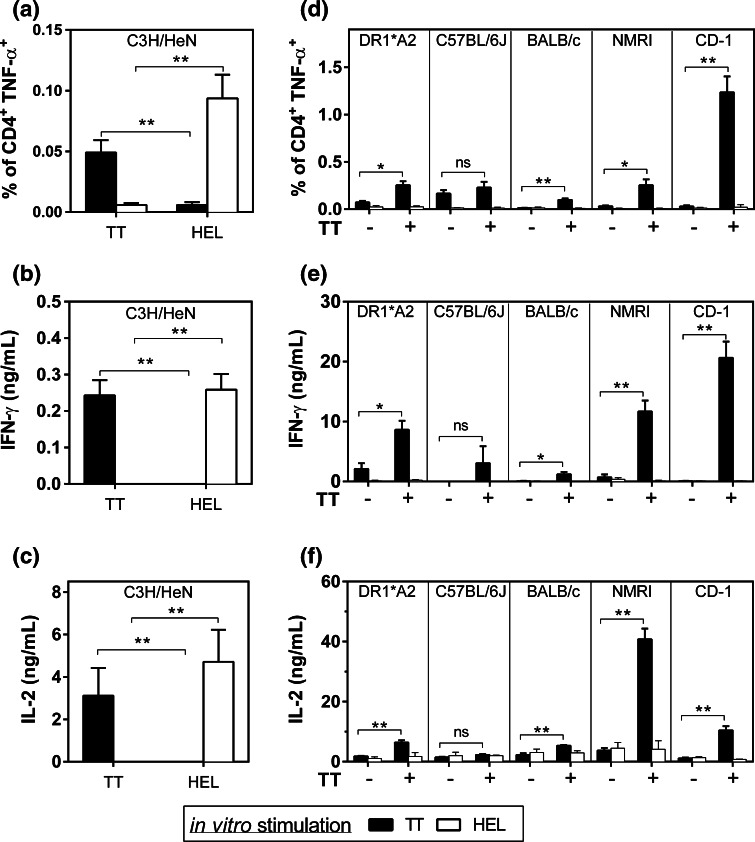
The TT epitope can be recognized and presented by different murine MHC class II and human HLA-DRB1*01:01 molecules. **a**–**c**. C3H/HeN mice (*n* = 5) were sc immunized with 50 μg of TT or HEL with CFA. On day 11, TNF-α production was analyzed by ICS on individual lymph nodes (**a**) and IFN-γ (**b**) and IL-2 (**c**) production was analyzed by ELISA on pooled lymph nodes stimulated with TT (*black bars*) or HEL (*white bars*). The results are expressed as the mean of individual mice ± SEM. **d, e, f.** HLA-DR1*A2 (*n* = 3), C57BL/6J (*n* = 3), BALB/c (*n* = 3), NMRI (*n* = 3) and CD-1 (*n* = 6) mice were sc immunized with 50 μg of TT or with immunostimulant alone on days 0 and 14. On day 28, TNF-α production was analyzed by ICS (**d**) and IFN-γ (**e**) and IL-2 (**f**) production was analyzed by ELISA on splenocytes stimulated with TT (*black bars*) or HEL (*white bars*). The results are expressed as the mean of triplicates ± SEM. The statistical significance of differences was determined by the Student *t* test (*P < 0.05, **P < 0.01)

Following immunization with either TT or HEL, CD4^+^ T cells from C3H/HeN lymph nodes were able to produce TNF-α, IL-2 and IFN-γ in response to in vitro TT or HEL stimulation, showing the specificity of the T cell response (Fig. 3a–c, Sup. Fig. 3a).

In correlation with the T cell response observed following MAG-Tn3 vaccination (Fig. 1), the highest cytokine response to TT was observed for the outbred mice immunized with TT, while a lower response was measured for HLA-DR1*A2, and no response was detected for C57BL/6J mice (Fig. 3d–f, Sup. Fig. 3b). In these conditions, we were able to detect a T cell response to TT in BALB/c splenocytes, though it was lower than for HLA-DR1*A2 and outbred strains.

### Characterization of HLA-DRB molecules able to present TT

**Fig. 4:**
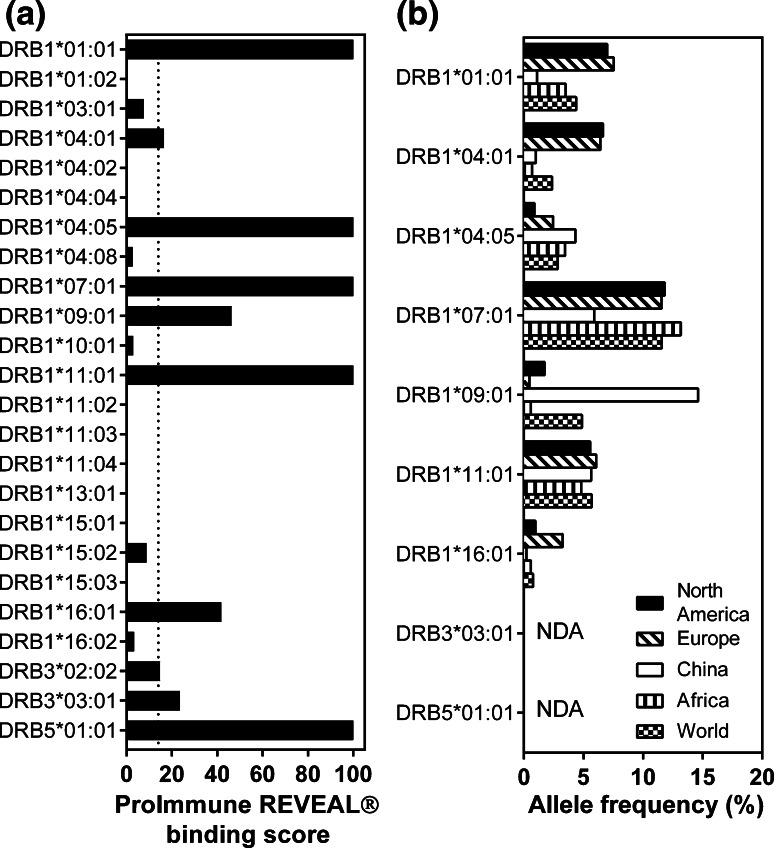
TT can be presented by different human HLA-DRB molecules. **a** The HLA binding assay was performed using TT against human HLA-DRB molecules of various HLA-DRB alleles. **b** The frequency of HLA-DRB molecules capable of binding TT is given for various populations, based on data published at www.allelefrequencies.net. *NDA* no data available

TT was initially defined as a promiscuous peptide due to its ability to induce the proliferation of human PBMCs expressing different HLA-DRB molecules [11–14, 28, 31], but its universal immunogenicity has recently been the subject of controversy [29, 32]. To have a more global view of TT immunogenicity in humans, we performed an HLA binding assay on HLA-DRB molecules corresponding to 24 human HLA-DRB alleles. We identified 9 HLA-DRBs capable of binding TT, with 100 % binding to HLA-DRB1*01:01, DRB1*04:05, DRB1*07:01, DRB1*11:01 and DRB5*01:01 (Fig. 4a). TT can be recognized by some of the most frequently expressed alleles, such as DR1*07:01, which is present in almost 15 % of all ethnic populations except Chinese, or DRB1*01:01 and DRB1*04:01, which are expressed by 6–8 % of North Americans and Europeans (Fig. 4b).

### Immunogenicity of the MAG-Tn3 vaccine in cynomolgus monkeys

**Fig. 5:**
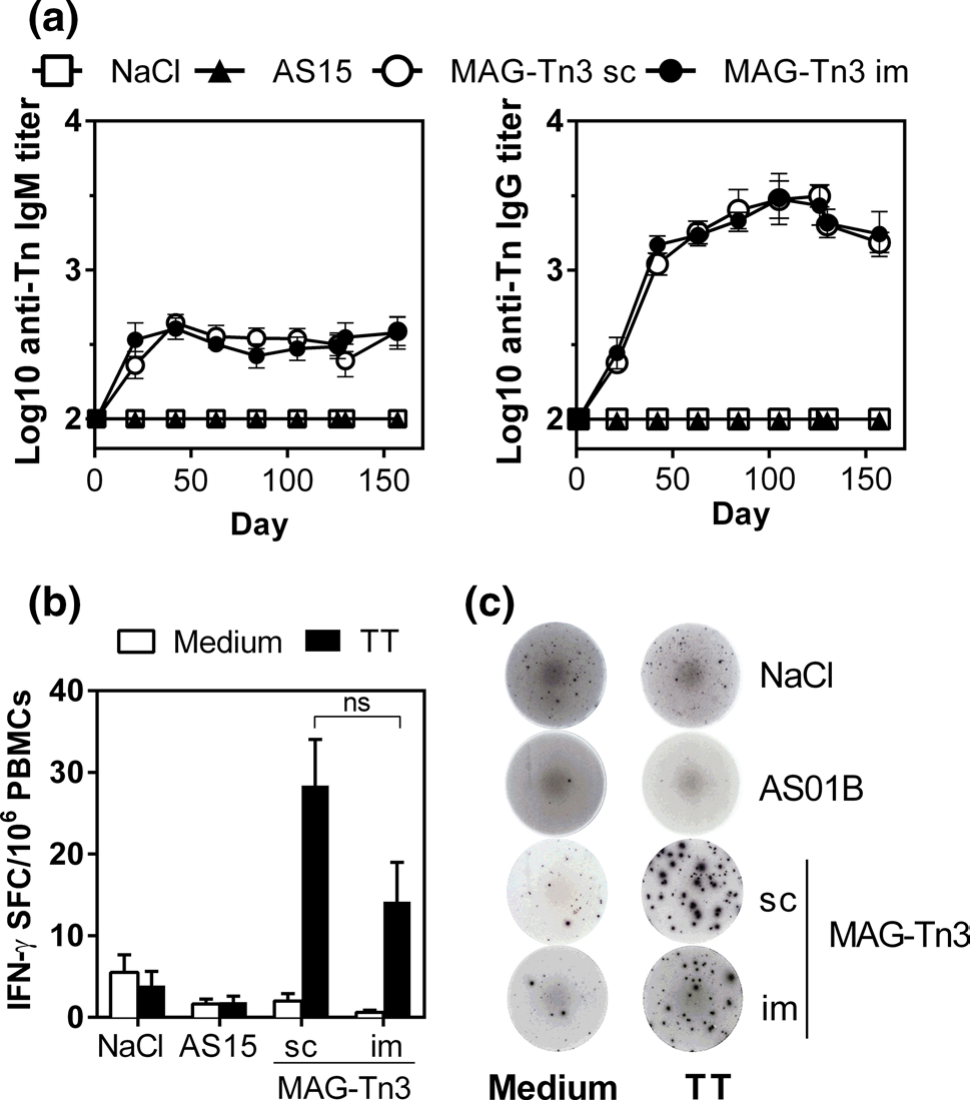
Immunization of cynomolgus monkeys with the MAG-Tn3 vaccine induces a specific anti-Tn antibody response associated with a T cell response to TT. Cynomolgus monkeys (*n* = 10/group) were immunized on days 0, 21, 42, 63, 84 and 105 with NaCl, AS15 or MAG-Tn3 formulated with AS15. **a** Sera were collected on the day of each immunization and tested for Tn-specific IgM and IgG responses by ELISA. **b**, **c** T cell responses were measured on cynomolgus PBMCs harvested after five immunizations (day 105), stimulated with (*black bars*) or without TT (*white bars*). IFN-γ production was evaluated by ELISPOT and is expressed as the mean of individual SFC per 10^6^ cells ± SEM (**b**). Pictures are shown of one representative well per group, seeded with 10^6^ cells (**c**). The statistical significance of differences was determined by the Student *t* test (**P < 0.01)

Finally, we evaluated MAG-Tn3 immunogenicity in a preclinical model of cynomolgus monkeys, which are more closely related to humans, using either sc or im routes of immunization. As shown in Fig. 5a, both anti-Tn IgM and IgG responses were detected in all animals immunized with the MAG-Tn3 vaccine, even after one immunization. This antibody response was associated with a T cell response to TT because cynomolgus PBMCs stimulated with TT were able to produce IFN-γ (Fig. 5b, c). No differences in immunogenicity were observed between the two routes of immunization.

**Fig. 6:**
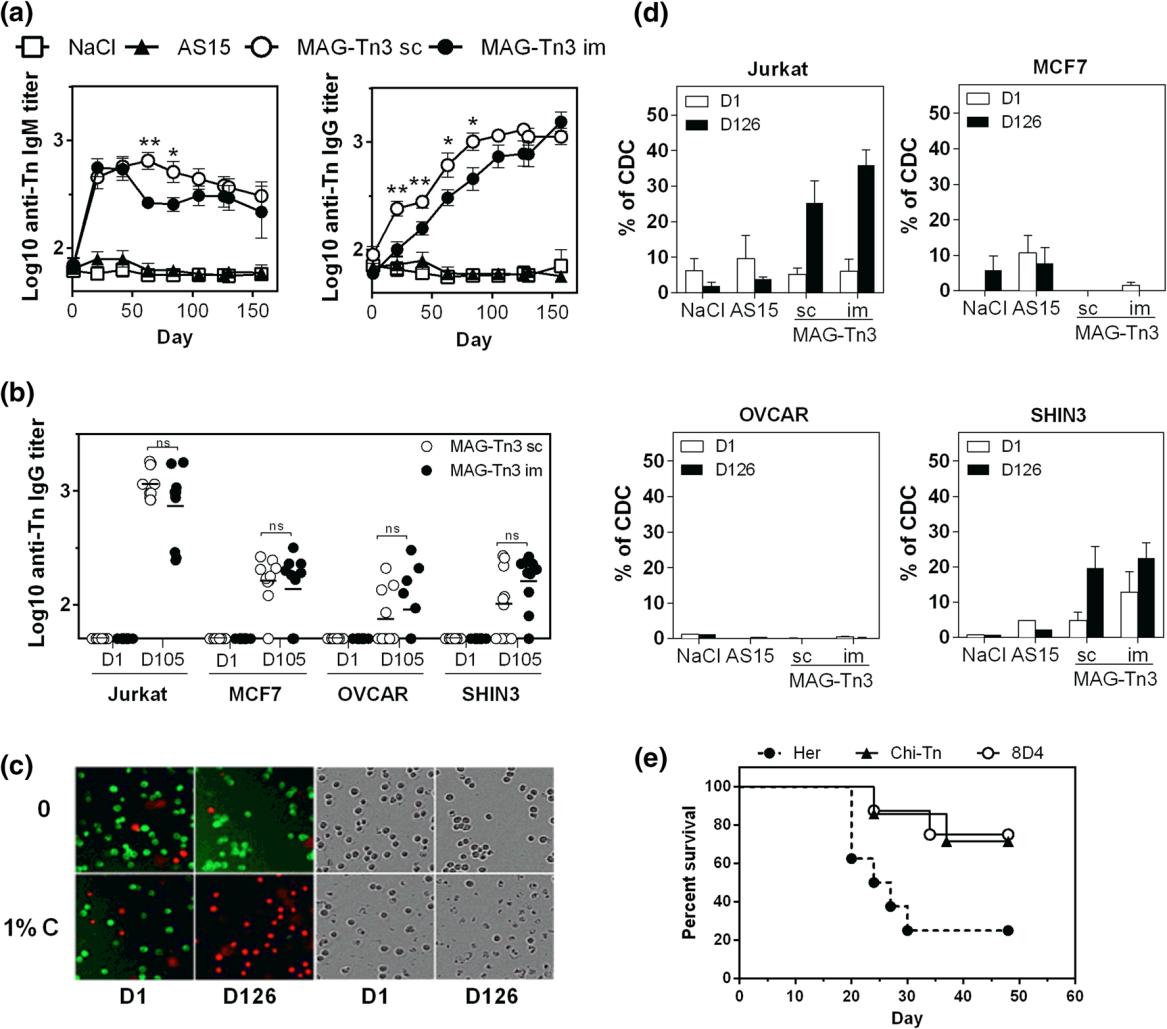
Anti-Tn antibodies are able to induce the death of Tn-expressing tumor cells in vitro and in vivo. **a**, **b.** Pre- and post-immunized sera, obtained from cynomolgus monkeys (*n* = 10/group) after the immunization protocol described in Fig. 5, were analyzed for their ability to recognize Tn-expressing tumor cells. The binding of sera to Jurkat cells was revealed using double labeling with anti-human-IgM-PE and anti-human-IgG-FITC (**a**). Sera of cynomolgus monkeys vaccinated with MAG-Tn3 were collected at day 105 and tested for Tn-specific binding to different Tn-expressing tumor cells, compared with naive sera (**b**). Antibody titers are expressed as the mean of Log10 individual antibody titers ± SEM. **c**, **d** CDC activity of pre-immunized (day 1) and post-immunized (day 126) sera of cynomolgus monkeys was analyzed on various Tn-expressing cells. Representative pictures of green and red fluorescence and associated transmission images for CDC on Jurkat-GFP cells are shown (**c**). The results are expressed as the mean of individual % of CDC ± SEM (**d**). **e** BALB/c mice grafted with TA3Ha cells were injected with CTX (50 mg/kg, day 1 after graft) and then treated (six injections) with the 8D4 murine anti-Tn mAb (*n* = 8), the Chi-Tn mAb (*n* = 8) or the Herceptin mAb (*n* = 6), and mouse survival was followed for 48 days. The statistical significance of differences was determined by the Student *t* test (*P < 0.05; **P < 0.01)

We then determined the biological activity of the anti-Tn antibodies produced in cynomolgus monkeys. In Fig. 6a, we showed that both anti-Tn IgM and IgG were able to recognize the human T-lymphoma Jurkat cells by FACS, and the anti-Tn antibody titers correlated with those determined by ELISA (Fig. 5a). We also observed recognition of the mammary carcinoma cell line MCF7 and the ovarian carcinoma cell lines SHIN3 and OVCAR (Fig. 6b and Sup. Fig. 4) to a lesser extent.

Because anti-Tn antibodies are able to target Tn-expressing tumor cells, we determined their ability to mediate killing of these cells through CDC activity (Fig. 6c, d). Sera of cynomolgus monkeys immunized with MAG-Tn3 were able to mediate CDC in Jurkat and SHIN3 cells, with no significant difference between the routes of immunization. Under these conditions, no killing of MCF7 and OVCAR cells was observed. No killing of Tn-expressing cells was observed with the sera of the NaCl or AS15 immunostimulant control groups (Fig. 6d). The CDC activity of cynomolgus antibodies was detectable at lower doses in Jurkat cells compared with SHIN3 cells (Sup. Fig. 5).

Finally, we analyzed the ability of anti-Tn antibodies to inhibit tumor growth in vivo by using the 8D4 murine mAb produced from MAG-Tn3-PV-treated mice, as previously described [3]. BALB/c mice grafted with the Tn-expressing TA3Ha murine breast cancer cells were treated with the 8D4 mAb, the chimeric mAb Chi-Tn [26], or with the irrelevant mAb Herceptin, in association with CTX treatment (Fig. 6e).

As previously described, treatment with the Chi-Tn mAb allowed 70 % of mice to survive [26], while mice treated with Herceptin had only 20 % survival. The mice treated with the 8D4 anti-Tn mAb displayed a survival percentage similar to mice treated with the Chi-Tn, demonstrating the protective capacity of the anti-Tn antibodies produced in response to MAG-Tn3 vaccination.

## Discussion

In the present study, we demonstrate that the MAG-Tn3 vaccine formulated with the AS15 immunostimulant is able to induce a high level of anti-Tn antibody production in both mice and cynomolgus monkeys, establishing the potential of the universal TT peptide as a T-helper epitope for the induction of anti-tumor immune responses against the Tn carcinoma antigen.

The MAG-Tn3 vaccine formulated in AS15 immunostimulant induced a strong anti-Tn IgG response in HLA-DR1*A2 transgenic mice, in C3H/HeN inbred mice and in two outbred strains of mice. This was associated with a T cell response oriented toward a T_H1_ profile and IFN-γ cytokine production, confirming that these mice were able to generate a CD4^+^ T cell response upon vaccination with MAG-Tn3, which helps B cells produce anti-Tn antibodies.

The ability to bind and present the TT peptide was confirmed through direct immunization assays. The T cell response intensity to direct TT immunization was correlated with the level of the anti-Tn antibody response to MAG-Tn3 in outbred and HLA-DR1*A2 mice. Interestingly, while C3H/HeN mice displayed high anti-Tn antibody response to MAG-Tn3, a weak T cell response to direct TT immunization was detected on lymph nodes. In addition, while BALB/c mice were able to respond to immunization with the TT peptide in CFA, confirming previous reports [8–10], no TT-specific T cell response nor anti-Tn antibody production was observed in these mice in response to vaccination by MAG-Tn3 in AS15. Thus, although the TT peptide can stimulate T cell responses in BALB/c mice in the presence of a strong adjuvant, its immunogenicity could not be sufficient to provide T cell help for anti-Tn antibody production after immunization with MAG-Tn3 in AS15.

Our results extended our knowledge on TT immunogenicity in mice because it can be presented by two outbred strains of mice, both belonging to the Swiss mouse family [33]. However, while these mice are currently randomly bred and thus constitute a heterogeneous population, their genetic heterogeneity depends on the stock history [33]. Costagliola et al. [34] determined that approximately 90 % of the NMRI mice used for their studies express the H-2^q^ haplotype, indicating reduced polymorphism for this strain. In contrast, Elia et al. [35] characterized greater polymorphism in a population of 30 CD-1 mice, in which mice expressed q/q (23 %), q/j (33 %), q/b (17 %), b/k (10 %) or j/j (17 %) haplotypes. Together, these data indicate that TT can be presented by H-2^q^ and H-2^j^ haplotypes.

TT was first described as a universal peptide based on its ability to be recognized by human PBMC expressing various HLA-DRB molecules, and to date, at least 10 different class II molecules have been described to be capable of interacting with TT [8, 12, 13, 15, 28, 29, 32]. However, these studies were performed using different types of assays. To have a more homogeneous analysis of TT immunogenicity in humans, we evaluated the ability of HLA-DRB molecules of 24 HLA-DRB alleles to bind this peptide using an HLA binding assay. We selected alleles of the three functional genes DRB1, DRB3 and DRB5 [36]. We mainly focused on DRB1 alleles because the DRB1 gene has the most polymorphisms; 1825 alleles have been reported so far [7]. The DRB1 allelic lineage is divided across five haplogroups: HLA-DRB1*01 and *10 (DR1), *08 (DR8), *15 and *16 (DR51), *03, *11, *13 and *14 (DR52) and finally *04, *07 and *09 (DR53) [37, 38]. We therefore selected the HLA-DRB1 alleles of various haplogroups.

We measured binding of TT by HLA-DRB1*01:01, DRB1*04:01, DRB1*11:01 and DRB3*03:01 and confirmed previous results [11–13, 28, 29, 32, 39]. We also confirmed the absence of binding by HLA-DRB1*15:01 [29]. However, in contrast to earlier studies, we were not able to detect the binding of TT by HLA-DRB1*03:01 and HLA-DRB1*11:02 [11, 12, 15, 32]. Finally, we characterized new HLA-DRB molecules that could bind TT: HLA-DRB1*04:05, DRB1*04:08, DRB1*07:01, DRB1*09:01 and DRB5*01:01. These results indicate that TT can bind to DRB1 molecules representative of four haplogroups (DRB1, DR51, DR52 and DR53).

MAG-Tn3/AS15 immunogenicity was finally evaluated in cynomolgus monkeys, which share with humans many similarities in their MHC II region. Indeed, similar to humans, the MHC II molecules of Old World monkeys (OWM), such as cynomolgus monkeys, are composed of multiple DRB regions [40, 41], with the presence of DRB1, DRB3 and DRB5 genes [42]. We previously showed that the MAG-Tn3 vaccine formulated in alum is able to induce an anti-Tn antibody response in African green and rhesus OWM species [30]. Here, we showed that the MAG-Tn3 vaccine formulated with the AS15 immunostimulant is able to induce anti-Tn antibody responses associated with an IFN-γ T cell response to TT in cynomolgus monkeys, and no adverse reactions were observed during the study (data not shown).

We showed that at least three different OWM species are able to respond to this peptide, reinforcing the universal nature of TT. However, it should be noted that, although OWM and humans have similarities in MHC class II, the DRB region of OWM is more complex, with both higher numbers and combinations of DRB genes than in humans, leading to the expression of more than 30 haplotypes [40, 41, 43, 44]. Moreover, some genes, named DRW genes, are specific to OWM, and configurations without DRB1 are found in OWM [44]. Finally, the allelic polymorphism of DRB1 genes seems to be limited compared with humans [36, 42].

This study also demonstrated the anti-tumor activity of the anti-Tn antibodies induced in response to MAG-Tn3 vaccination. Similar to other previously described anti-Tn antibodies, such as the therapeutic mAb MLS128 [45], these antibodies were able to recognize various Tn-expressing tumor cells, such as Jurkat and MCF7 tumor cell lines. The anti-tumor therapeutic potential of the antibodies elicited in cynomolgus monkeys was shown by their in vitro capacity to mediate the killing of Jurkat cells and SHIN3 ovarian carcinoma cells in the presence of complement. No killing of mammary MCF7 and ovarian OVCAR cell lines by CDC mechanism was observed. However, antibodies specific for tumor-associated antigens can also mediate tumor cell killing through the antibody-dependent cytotoxicity (ADCC) mechanism. Indeed, we previously showed that anti-Tn antibodies produced in rhesus monkeys in response to MAG-Tn3 formulated in alum were able to mediate the killing of Jurkat cells in the presence of human NK cells [30]. Finally, the 8D4 murine anti-Tn mAb, in combination with CTX treatment, allowed for the survival of 70 % of TA3Ha tumor-bearing mice, demonstrating the therapeutic potential of anti-Tn antibodies in vivo.

In conclusion, this preclinical study confirmed the strong potential of the MAG-Tn3 vaccine formulated with AS15 for the induction of functional antibody responses against the tumor-specific Tn antigen. This vaccine candidate is currently under evaluation in a phase I clinical trial in breast cancer patients.

### Electronic supplementary material

Below is the link to the electronic supplementary material.

Supplementary material 1 (PDF 770 kb)

## Abbreviations

ADCC: Antibody-dependent cytotoxicity
CDC: Complement-dependent cytotoxicity
CETEA: Comité d’éthique en expérimentation animale
CFA: Complete Freund’s adjuvant
CFSE: Carboxyfluorescein succinimidyl ester
CTX: Cyclophosphamide
GalNac: N-acetylgalactosamine
GMP: Good manufacturing practices
ICS: Intracellular staining
IFA: Incomplete Freund’s adjuvant
im: Intramuscular/intramuscularly
ip: Intraperitoneal/intraperitoneally
mAb: Monoclonal antibody
MAG: Multiple antigenic glycopeptide
ODN: Oligodeoxynucleotides
OWM: Old World monkeys
PV: Poliovirus
sc: Subcutaneous/subcutaneously
SFC: Spot-forming cells
TT: TT830-844 peptide
