# Supplementary material for: The fully synthetic MAG-Tn3 therapeutic vaccine containing the tetanus toxoid-derived TT830-844 universal epitope provides anti-tumor immunity
Source: Cancer Immunol Immunother. 2016 Feb 4;65(3):315–25. doi: 10.1007/s00262-016-1802-0 (PMC4779142; doi:10.1007/s00262-016-1802-0)
Supplement: Supplementary file 1 — Supplementary material 1 (PDF 770 kb) [file 262_2016_1802_MOESM1_ESM.pdf]

Supplementary Figure 1

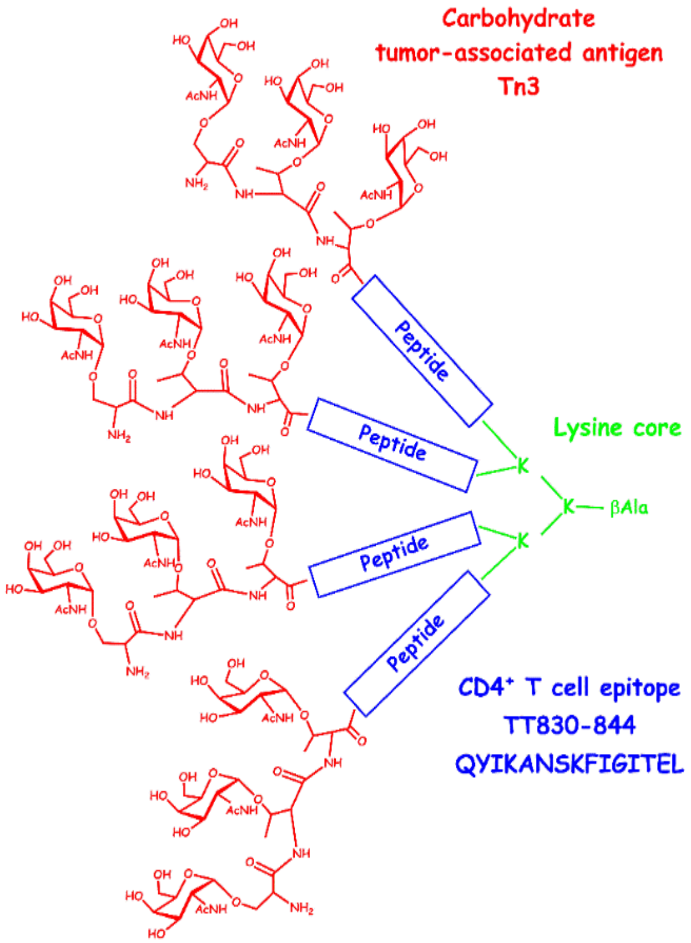

Schematic representation of the MAG-Tn3 vaccine. MAG-Tn3 is composed of a lysine core carrying four copies of the TT peptide further extended with a trimer of the Tn antigen.

Supplementary Figure 2

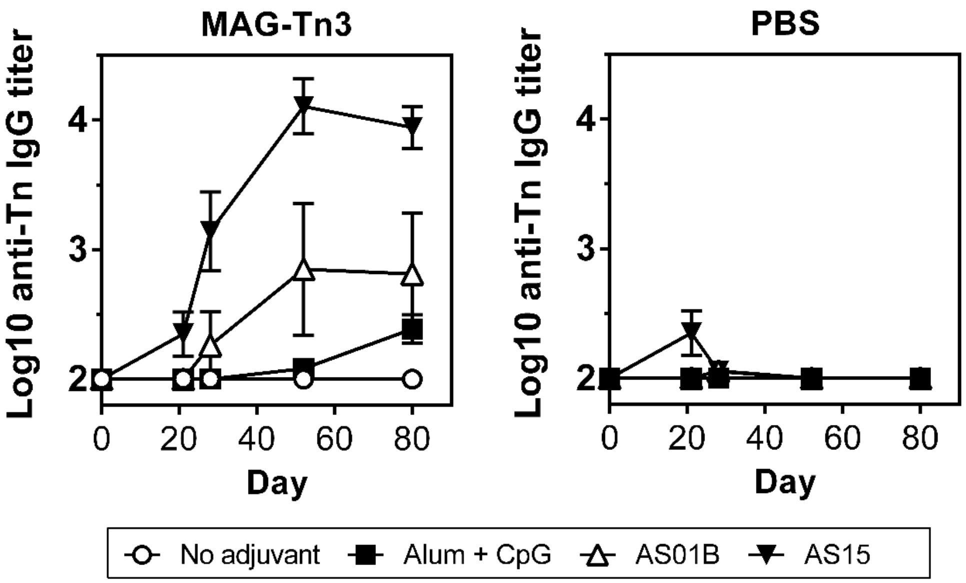

The AS15 immunostimulant allows high anti-Tn antibody production in response to the MAG-Tn3 vaccine. HLA-DR1*A2 mice (n=4/group) were sc immunized with 10 µg of MAG-Tn3 (left panel) in association with Alum + CpG (1 mg Alum + 10 µg CpG 1826), AS01B (1/10 human dose) or AS15 immunostimulant (1/10 human dose), or with immunostimulant alone (right panel), on days 0, 21, 45 and 70. AS01B is a combination of 3-O-desacyl-4'-monophosphoryl lipid A (MPL, 50 µg, produced by GSK) and QS-21 (50 µg, Quillaja saponaria Molina, fraction 21 [Antigenics Inc, a wholly owned subsidiary of Agenus Inc., Lexington, MA, USA]) in a liposomal formulation.

Sera were collected on days 21, 28, 52 and 80 and tested for Tn-specific IgG by ELISA using Tn3-G6K(Biot)G. Results are representative of two experiments. Antibody titers are expressed as the mean of Log10 individual antibody titers ± SEM.

Supplementary Figure 3

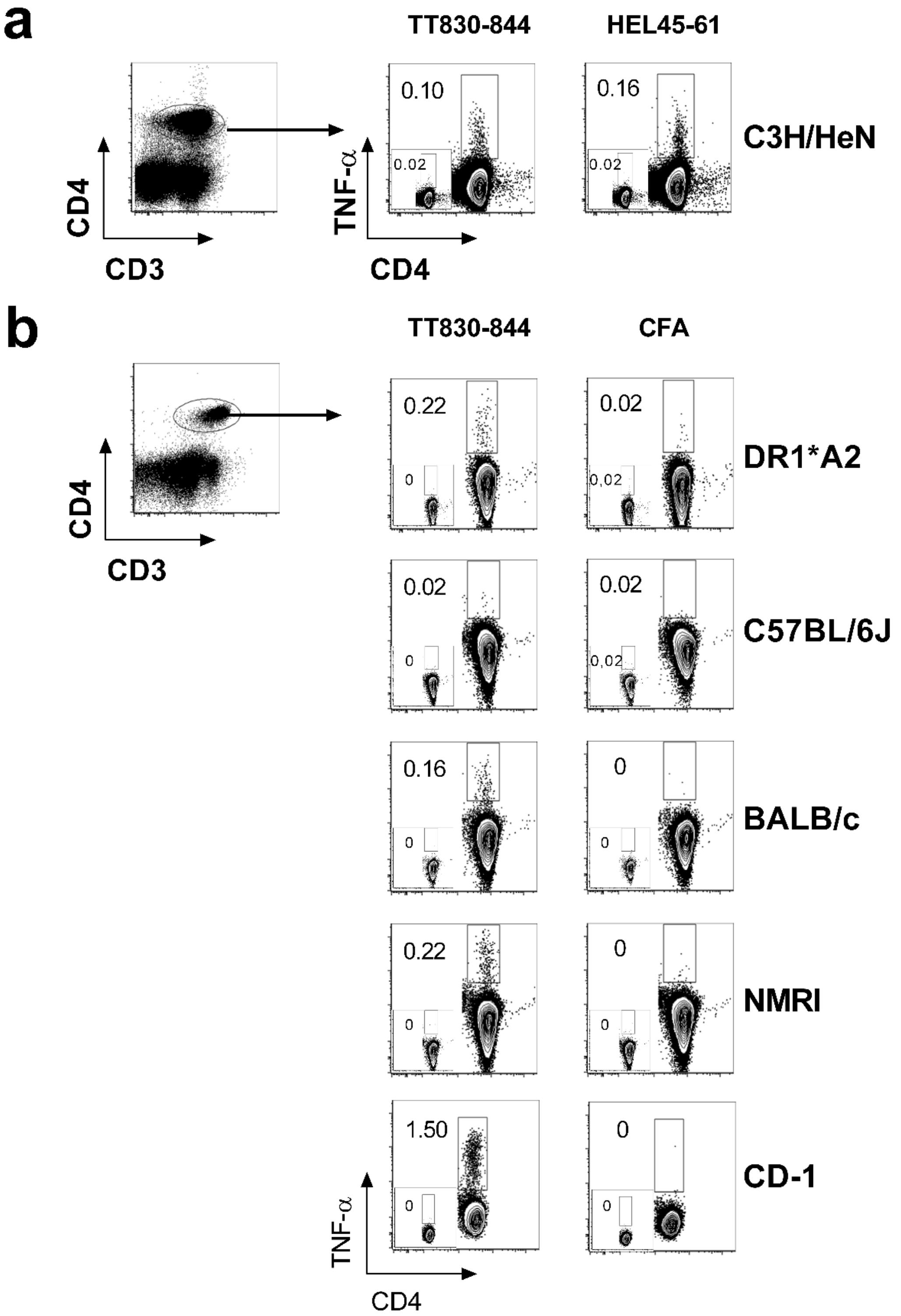

Dot plots of TNF-α response after stimulation with TT measured by ICS. TNF-α production by $CD3^+CD4^+$ cells was analyzed by ICS on mice immunized with either TT or HEL peptide in CFA, or with adjuvant alone, as detailed in Fig. 3, after 2 hours stimulation with 50 µg/mL of TT or HEL in the presence of BFA, on individual lymph nodes (a) or spleens (b). Dot plots of one representative lymph node or spleen stimulated with specific peptide (large square) or non-specific peptide (small square) are shown.

Supplementary Figure 4

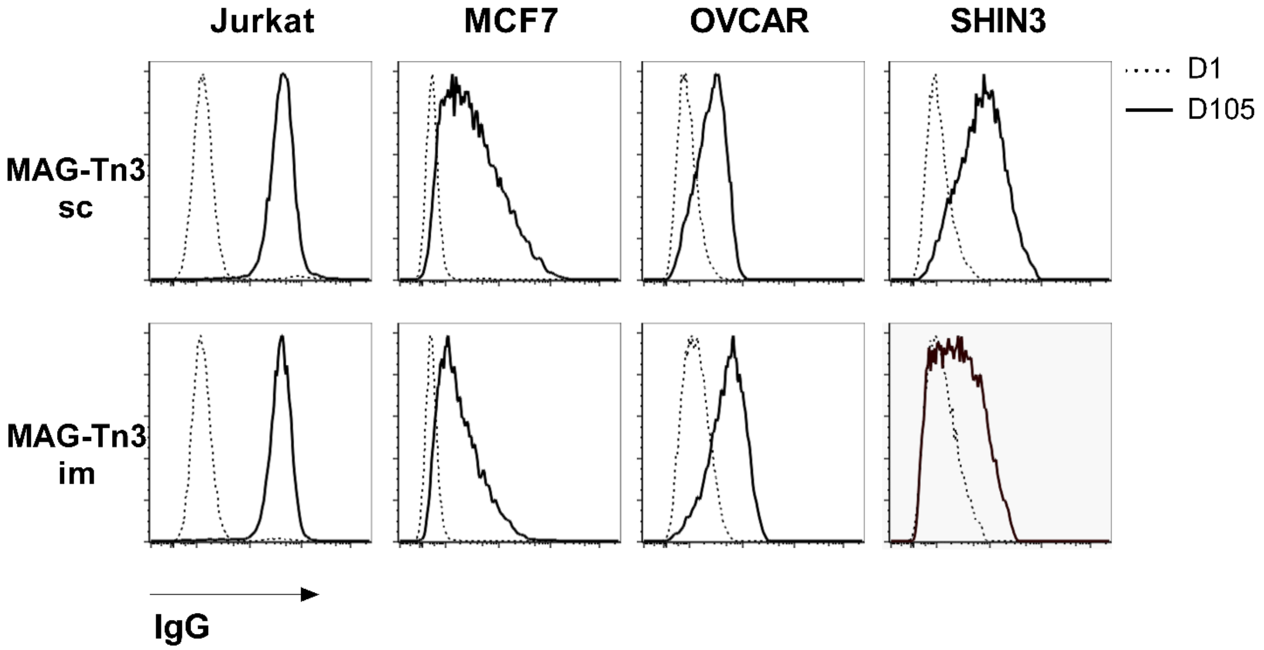

Recognition of Tn-expressing tumor cells by sera of cynomolgus monkeys immunized with the MAG-Tn3 vaccine. Pre- (Day 1) and post-immune (Day 105) sera of cynomolgus monkeys obtained after the immunization protocol detailed in Fig. 5 were analyzed for anti-Tn IgM and IgG responses against different Tn-expressing tumor cells as showed in Fig. 6a and b. FACS histograms of representative pre-immune (dashed lines) and post-immune sera (bold line) of cynomolgus monkeys immunized with the MAG-Tn3 vaccine are shown for each Tn-expressing tumor cell line tested.

Supplementary Figure 5

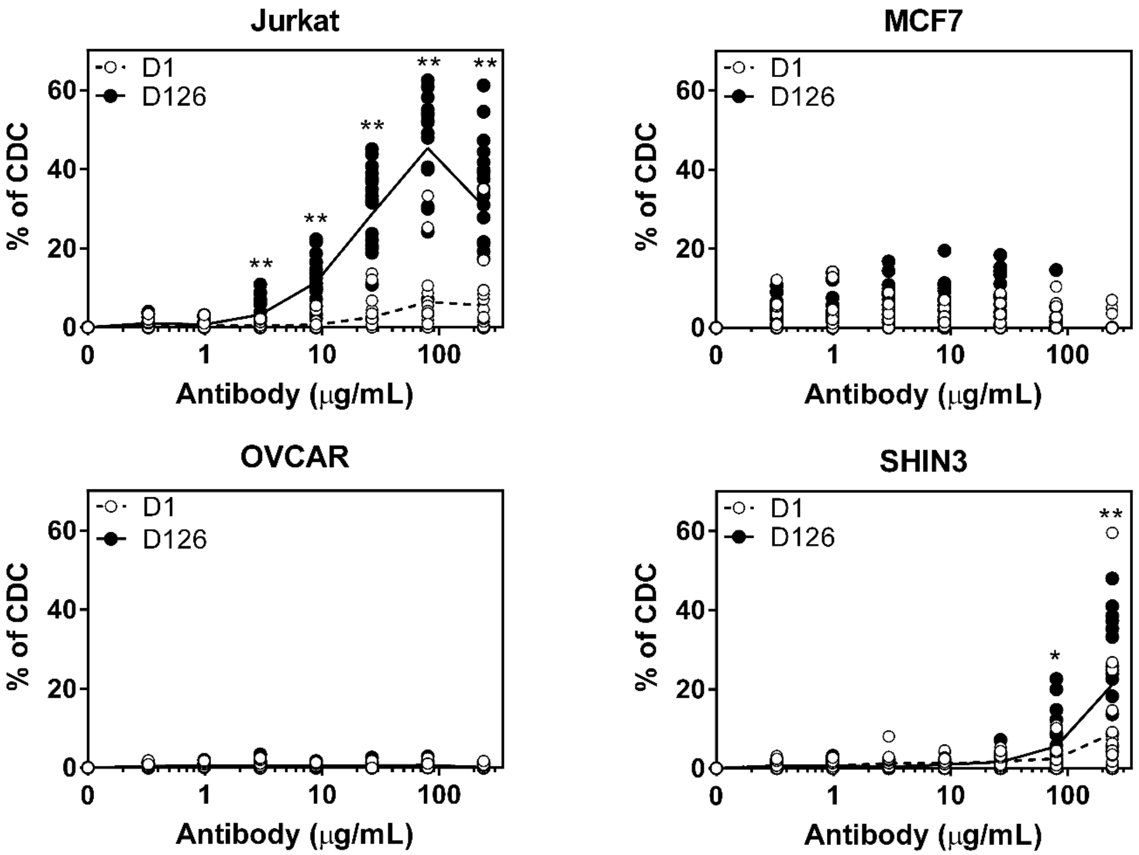

Dose-dependent CDC activity of antibodies from sera of cynomolgus monkeys immunized with the MAG-Tn3 vaccine on different Tn-expressing tumor cells. Tn-positive cells expressing GFP or CFSE-stained, were incubated with various concentrations of IgG purified from pre- (Day 1) and post-immune (Day 126) sera of cynomolgus monkeys obtained after the immunization protocol detailed in Fig. 5, in the presence of rabbit complement and DRAQ7 as viability marker. After 2 hours incubation (37°C), the numbers of live (green fluorescent) and dead cells (red fluorescent) were measured by microscopic analysis with Incucyte Instrument. Dose dependent CDC activity on Tn-expressing cells is shown. The statistical significance of differences was determined by the Student t test (** $P<0.01$).
